# Crystal structure of trirubidium citrate monohydrate from laboratory X-ray powder diffraction data and DFT comparison

**DOI:** 10.1107/S2056989017000743

**Published:** 2017-01-20

**Authors:** Alagappa Rammohan, James A. Kaduk

**Affiliations:** a Atlantic International University, Honolulu, HI, USA; b Illinois Institute of Technology, Chicago, IL, USA

**Keywords:** crystal structure, powder diffraction, density functional theory, citrate, rubidium

## Abstract

The crystal structure of the title compound, 3Rb^+^·C_6_H_5_O_7_^3−^·H_2_O, has been solved and refined using laboratory X-ray powder diffraction data, and optimized using density functional techniques. The hy­droxy group participates in an intra­molecular hydrogen bond to the deprotonated central carboxyl­ate group with graph-set motif *S*(5). The water mol­ecule acts as a hydrogen-bond donor to both terminal and central carboxyl­ate O atoms. The three independent rubidium cations are seven-, six- and six-coordinate, with bond-valence sums of 0.84, 1.02, and 0.95, respectively. In the extended structure, their polyhedra share edges and corners to form a three-dimensional network. The hydro­phobic methyl­ene groups occupy channels along the *b* axis.

## Abstract 2

The crystal structure of trirubidium citrate monohydrate has been solved and refined using laboratory X-ray powder diffraction data, and optimized using density functional techniques.

## Chemical context   

In the course of a systematic study of the crystal structures of Group 1 (alkali metal) citrate salts to understand the anion’s conformational flexibility, deprotonation, coordination tendencies, and hydrogen bonding, we have determined several new crystal structures. Most of the new structures were solved using powder diffraction data (laboratory and/or synchrotron), but single crystals were used where available. The general trends and conclusions about the sixteen new compounds and twelve previously characterized structures are being reported separately (Rammohan & Kaduk, 2017*a* ▸). Seven of the new structures – NaKHC_6_H_5_O_7_, NaK_2_C_6_H_5_O_7_, Na_3_C_6_H_5_O_7_, NaH_2_C_6_H_5_O_7_, Na_2_HC_6_H_5_O_7_, K_3_C_6_H_5_O_7_, and Rb_2_HC_6_H_5_O_7_ – have been published recently (Rammohan & Kaduk, 2016*a* ▸,*b* ▸,*c* ▸,*d* ▸,*f* ▸, 2017*b* ▸; Rammohan *et al.* (2016 ▸)), and two additional structures – KH_2_C_6_H_5_O_7_ and KH_2_C_6_H_5_O_7_(H_2_O)_2_ – have been communicated to the CSD (Kaduk & Stern, 2016**a* ▸,b* ▸).
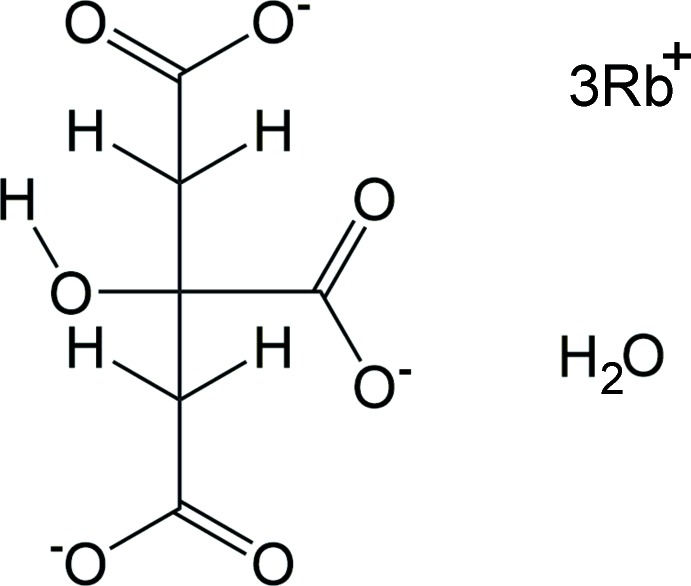


## Structural commentary   

**Figure 1:**
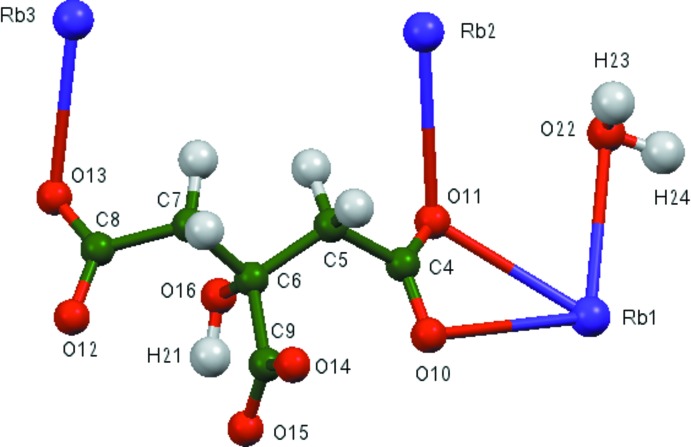
The asymmetric unit, with the atom numbering. The atoms are represented by 50% probability spheroids.

**Figure 2:**
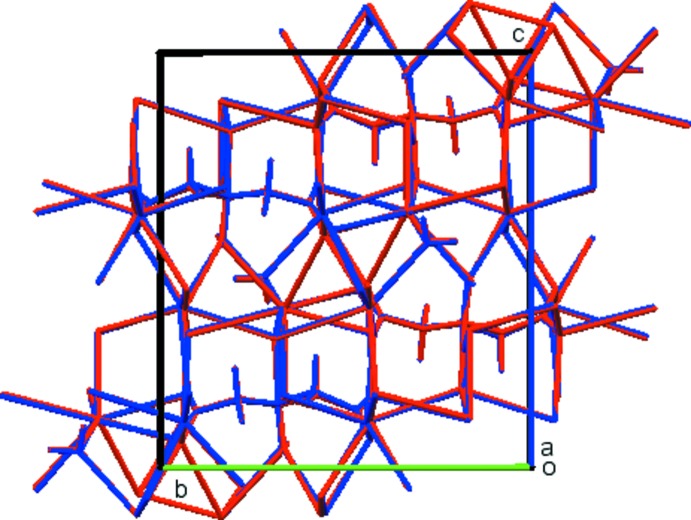
Comparison of the refined and optimized structures of trirubidium citrate monohydrate. The refined structure is in red, and the DFT-optimized structure is in blue.

The asymmetric unit of the title compound is shown in Fig. 1 ▸. The root-mean-square deviation of the non-hydrogen atoms in the Rietveld-refined and DFT-optimized structures is 0.127 Å (Fig. 2 ▸). The good agreement between the two structures is strong evidence that the experimental structure is correct (van de Streek & Neumann, 2014 ▸). This discussion uses the DFT-optimized structure. Most of the bond lengths, bond angles, and torsion angles fall within the normal ranges indicated by a *Mercury* Mogul geometry check (Macrae *et al.*, 2008 ▸). Only the O11—C4—C5—C6 torsion angle involving a terminal carboxyl­ate group is flagged as unusual, but as shown in Rammohan & Kaduk (2017*a* ▸) these torsion angles exhibit no real preference. The citrate anion occurs in the *trans,trans*-conformation, which is one of the two low-energy conformations of an isolated citrate trianion. The central carboxyl­ate group and the hydroxyl group occur in the normal planar arrangement. The terminal carboxyl­ate O13 atom and the hy­droxy group O16 atom chelate to Rb3. The terminal carboxyl­ate O12 atom and the central carboxyl­ate O15 atom chelate to another Rb3 cation. The terminal carboxyl­ate O12 and central carboxyl­ate O14 chelate to Rb2, and the terminal O10 and central O14 chelate to a third Rb3 atom. The terminal carboxyl­ate O14/O15 acts as a bidentate ligand to Rb1, and the terminal carboxyl­ate O10/O11 chelates to another Rb1.

The Bravais–Friedel–Donnay–Harker (Bravais, 1866 ▸; Friedel, 1907 ▸; Donnay & Harker, 1937 ▸) morphology suggests that we might expect platy morphology for the title compound, with {011} as the principal faces. A 4th-order spherical harmonic texture model was included in the refinement. The texture index was 1.014, indicating that preferred orientation was negligible for this rotated flat-plate specimen.

## Supra­molecular features   

**Figure 3:**
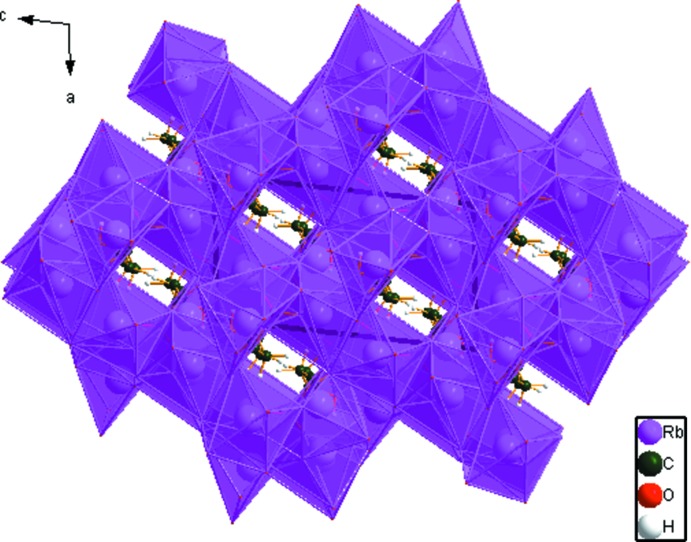
Crystal structure of trirubidium citrate monohydrate, viewed down the *b* axis. outline of the unit cell needs to be added

The three independent Rb^+^ ions are 7-, 6- and 6-coordinate (upper threshold for Rb—O bond lengths = 3.40 Å), with bond-valence sums of 0.84, 1.02, and 0.95, respectively. These polyhedra share edges and corners to form a three-dimensional network (Fig. 3 ▸). Hydrogen bonds (Table 1 ▸) between the water mol­ecules and the citrate anions result in chains propagating along the *b*-axis direction. The hydroxyl group participates in an intra­molecular hydrogen bond to the deprotonated central carboxyl­ate group with graph-set motif *S*(5). The water mol­ecule acts as a hydrogen-bond donor to both the terminal carboxyl­ate atom O13 and the central carboxyl­ate atom O14. The Mulliken overlap populations indicate, by the correlation in Rammohan & Kaduk (2017*a* ▸), that these hydrogen bonds account for 41.6 kcal mol^−1^ of crystal energy. A C—H⋯O hydrogen bond also apparently contributes to the crystal energy. The hydro­phobic methyl­ene groups occupy channels along the *b*-axis. This compound is isostructural to K_3_C_6_H_5_O_7_(H_2_O) (Carrell *et al.*, 1987 ▸; CSD Refcode ZZZHVI01).

## Database survey   

Details of the comprehensive literature search for citrate structures are presented in Rammohan & Kaduk (2017*a* ▸). A reduced cell search of the cell of trirubidium citrate monohydrate in the Cambridge Structural Database (Groom *et al.*, 2016 ▸) (increasing the default tolerance from 1.5 to 2.0%) yielded 228 hits, but combining the cell search with a citrate fragment yielded Love & Patterson (1960 ▸, CSD Refcode ZZZHZC), but no coordinates were reported for this phase. Increasing the tolerance on the cell to 5% yielded K_3_C_6_H_5_O_7_(H_2_O) (Burns & Iball, 1954 ▸, CSD Refcode ZZZHVI; Carrell *et al.*, 1987 ▸, CSD Refcodes ZZZHVI01 and ZZZHVI02).

## Synthesis and crystallization   

H_3_C_6_H_5_O_7_(H_2_O) (10.0 mmol, 2.0972 g) was dissolved in 10 ml deionized water. Rb_2_CO_3_ (15.0 mmol, 3.4659 g, Sigma–Aldrich) was added to the citric acid solution slowly with stirring. The resulting clear colourless solution was evaporated to dryness at ambient conditions to yield a white powder.

## Refinement   

**Figure 4:**
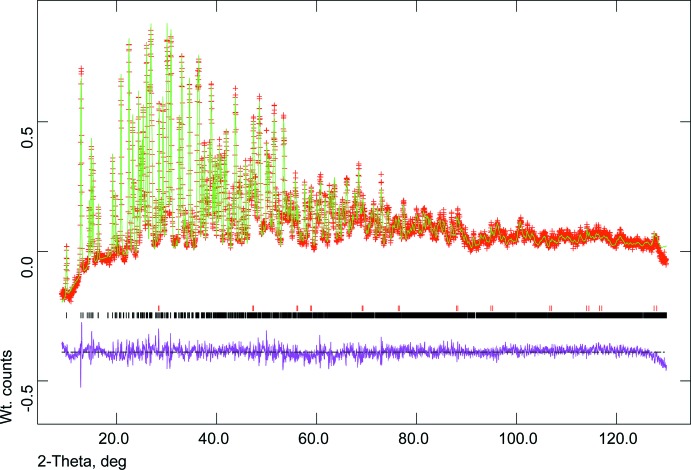
Rietveld plot for the refinement of trirubidium citrate monohydrate. The vertical scale is not the raw counts but the counts multiplied by the least squares weights. This plot emphasizes the fit of the weaker peaks. The red crosses represent the observed data points, and the green line is the calculated pattern. The magenta curve is the difference pattern, plotted at the same scale as the other patterns. The row of black tick marks indicates the reflection positions, and the red tick marks indicate the Si inter­nal standard peak positions.

Crystal data, data collection and structure refinement details are summarized in Table 2 ▸. The specimen was blended with a NIST SRM 640 Si inter­nal standard (*a* = 5.43105 Å). The powder pattern (Fig. 4 ▸) was indexed using *Jade 9.4* (MDI, 2012 ▸), which yielded a primitive monoclinic cell having *a* = 7.44769 (10), *b* = 11.87554 (16), *c* = 13.41675 (18) Å, β = 97.8820 (9)°, *V* = 1175.44 (3) Å^3^, and *Z* = 4. The suggested space group was *P*2_1_/*n*, which was confirmed by successful solution and refinement. Three intense peaks from a structure solution using charge flipping as implemented in *Jana2006* (Petříček *et al.*, 2014 ▸) were used to carry out a Le Bail fit in *GSAS* (Larson & Von Dreele, 2004 ▸). The resulting peak list was imported into *Endeavour 1.7b* (Putz *et al.*, 1999 ▸), which was used to solve the structure with a citrate anion and 3 Rb atoms as fragments. A significant peak in a difference Fourier map in *GSAS* corresponded to the oxygen atom of a water mol­ecule, indicating that the compound was a monohydrate.

Pseudo-Voigt profile coefficients were as parameterized in Thompson *et al.* (1987 ▸) with profile coefficients for Simpson’s rule integration of the pseudo-Voigt function according to Howard (1982 ▸). The asymmetry correction of Finger *et al.* (1994 ▸) was applied, and microstrain broadening by Stephens (1999 ▸). The structure was refined by the Rietveld method using *GSAS/EXPGUI* (Larson & Von Dreele, 2004 ▸; Toby, 2001 ▸). All C—C and C—O bond lengths were restrained, as were all bond angles. The hydrogen atoms were included at fixed positions, which were recalculated during the course of the refinement using *Materials Studio* (Dassault Systemes, 2014 ▸). The *U*_iso_ values of the C and O atoms in the citrate anion were constrained to be equal, and the *U*_iso_ values of the hydrogen atoms were constrained to be 1.3 times those of the atoms to which they are attached.

## DFT calculations   

After the Rietveld refinement, a density functional geometry optimization (fixed experimental unit cell) was carried out using *CRYSTAL09* (Dovesi *et al.*, 2005 ▸). The basis sets for the C, H, and O atoms were those of Gatti *et al.* (1994 ▸), and the basis set for Rb was that of Schoenes *et al.* (2008 ▸). The calculation used 8 *k*-points and the B3LYP functional, and took about 72 h on a 2.4 GHz PC. The *U*_iso_ values from the Rietveld refinement were assigned to the optimized fractional coord­inates.

## Supplementary Material

Crystal structure: contains datablock(s) RAMM010_publ, ramm010_DFT, RAMM010_overall, RAMM010_phase_1, RAMM010_phase_2, RAMM010_p_01. DOI: 10.1107/S2056989017000743/hb7648sup1.cif

CCDC references: 1527729, 1527730, 1527731

Additional supporting information:  crystallographic information; 3D view; checkCIF report

## Figures and Tables

**Table 1:** Hydrogen-bond geometry (Å, °)

*D*—H⋯*A*	*D*—H	H⋯*A*	*D*⋯*A*	*D*—H⋯*A*
O16—H21⋯O15	0.984	1.838	2.552	126.9
O22—H23⋯O14^i^	0.983	1.704	2.672	168.7
O22—H24⋯O13	0.984	1.707	2.683	170.8
C5—H17⋯O22	1.093	2.674	3.749	167.4

Symmetry code: (i) 
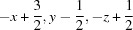
.

**Table 2:** Experimental details

	Powder data
Crystal data	
Chemical formula	3Rb^+^·C_6_H_5_O_7_^3−^·H_2_O
*M*_r_	463.52
Crystal system, space group	Monoclinic, *P*2_1_/*n*
Temperature (K)	300
*a*, *b*, *c* (Å)	7.44769 (10), 11.87554 (16), 13.41675 (18)
β (°)	97.8820 (9)
*V* (Å^3^)	1175.44 (4)
*Z*	4
Radiation type	*K*α_1_, *K*α_2_, λ = 1.540629, 1.544451 Å
Specimen shape, size (mm)	Flat sheet, 24 × 24

Data collection	
Diffractometer	IIT Bruker D2 Phaser
Specimen mounting	Si zero-background cell
Data collection mode	Reflection
Scan method	Step
2θ values (°)	2θ_min_ = 5.042 2θ_max_ = 130.045 2θ_step_ = 0.020

Refinement	
*R* factors and goodness of fit	*R*_p_ = 0.015, *R*_wp_ = 0.019, *R*_exp_ = 0.007, *R*(*F* ^2^) = 0.061, χ^2^ = 8.352
No. of parameters	88
No. of restraints	47

The same symmetry and lattice parameters were used for the DFT calculation. Computer programs: *DIFFRAC.Measurement* (Bruker, 2009 ▸), (*GSAS*, Larson & Von Dreele, 2004 ▸), *DIAMOND* (Crystal Impact, 2015 ▸) and *publCIF* (Westrip, 2010 ▸).

## supplementary crystallographic information

### Crystal data

**Table:**

3Rb^+^·C_6_H_5_O_7_^3^^−^·H_2_O	β = 97.8820°
*M**_r_* = 463.48	*V* = 1175.44 Å^3^
Monoclinic, *P*2_1_/*n*	*Z* = 4
*a* = 7.4477 Å	Cu *K*α radiation, λ = 1.5418 Å
*b* = 11.8755 Å	*T* = 300 K
*c* = 13.4168 Å	

### Data collection

**Table:**

Density functional calculation	*k* = →
*h* = →	*l* = →

### Fractional atomic coordinates and isotropic or equivalent isotropic displacement parameters (Å^2^)

**Table:**

	*x*	*y*	*z*	*U*_iso_*/*U*_eq_	
Rb1	0.00583	0.17144	0.11496	0.04560*	
Rb2	0.11938	0.43645	0.38752	0.04560*	
Rb3	0.33332	0.44502	0.10955	0.04560*	
C4	0.88224	0.90099	0.13558	0.03270*	
C5	0.79490	0.79062	0.16279	0.03270*	
C6	0.90539	0.68265	0.15421	0.03270*	
C7	0.78654	0.58443	0.18427	0.03270*	
C8	0.83169	0.47105	0.13960	0.03270*	
C9	1.09412	0.68118	0.22083	0.03270*	
O10	1.03887	0.92354	0.18002	0.03510*	
O11	0.78874	0.96367	0.07211	0.03510*	
O12	0.97854	0.42387	0.17390	0.03510*	
O13	0.71389	0.43264	0.07008	0.03510*	
O14	1.10018	0.68748	0.31550	0.03510*	
O15	1.22967	0.66839	0.17504	0.03510*	
O16	0.93669	0.67035	0.05167	0.03510*	
H17	0.66841	0.77938	0.11192	0.04250*	
H18	0.76254	0.79781	0.23982	0.04250*	
H19	0.64492	0.60516	0.15820	0.04250*	
H20	0.80258	0.57869	0.26635	0.04250*	
H21	1.06826	0.65697	0.05902	0.04560*	
O22	0.60639	0.21834	0.03896	0.03990*	
H23	0.54454	0.20759	0.09843	0.05180*	
H24	0.65210	0.29614	0.04430	0.05180*	

### Bond lengths (Å)

**Table:**

C4—C5	1.530	C7—H19	1.093
C4—O10	1.264	C7—H20	1.093
C4—O11	1.266	C8—O12	1.258
C5—C6	1.536	C8—O13	1.274
C5—H17	1.093	C9—O14	1.267
C5—H18	1.096	C9—O15	1.261
C6—C7	1.551	O16—H21	0.984
C6—C9	1.559	O22—H23	0.983
C6—O16	1.434	O22—H24	0.984
C7—C8	1.530		

### Hydrogen-bond geometry (Å, º)

**Table:**

*D*—H···*A*	*D*—H	H···*A*	*D*···*A*	*D*—H···*A*
O16—H21···O15	0.984	1.838	2.552	126.9
O22—H23···O14^i^	0.983	1.704	2.672	168.7
O22—H24···O13	0.984	1.707	2.683	170.8
C5—H17···O22	1.093	2.674	3.749	167.4

Symmetry code: (i) −*x*+3/2, *y*−1/2, −*z*+1/2.
